# To the Editors of the Medical and Physical Journal

**Published:** 1804-11-01

**Authors:** Samuel Hill

**Affiliations:** Surgeon, of Queen Street, Town of Portsea, and Surgeon in the Royal Navy


### *To the Editors of the Medical and Physical Journal.*

GENTLEMEN,

I AM sorry to see that Dr. Macdonald, in his observations on Mr. Goldson's Pamphlet, has committed himself by introducing me, as hostile to vacciolation: I conceive there is considerable indelicacy in thus attacking a person, whose sentiments he most certainly does not know. I shall not degrade myself, nor the character of my profession, by answering him in the same style in which he has chosen to use my name*; I think it sufficient for me to observe, that the conclusion he has drawn, from seeing my name in the pamphlet above alluded to, is diametrically opposite to the truth; I however, confess, that I would rather Dr. Macdonald should be in an error, than I an enemy to the new practice. Gentlemen should support their opinions by a temperate relation of facts, and fair dispassionate argument, and should on every occasion, let the difference in their sentiments be ever so great, treat each other with becoming respect.

By

---

* See London Medical and Physical Journal for October, pages 314 and 316.

By giving this letter a place in your Journal for the ensuing month, you will greatly oblige,

Yours, &c.

*October* 9, 1804. SAMUEL HILL,

Surgeon, of Queen Street, Town of Portsea, and Surgeon in the Royal Navy.

---

### *To the Editors of the Medical and Physical Journal.*

GENTLEMEN,

